# Compartment-specific transcriptional programs of the gravid human myometrium underlie labor and uterine atony

**DOI:** 10.21203/rs.3.rs-11225136/v1

**Published:** 2026-10-23

**Authors:** David Cornfield, Jessica Ansari, Daiana Fornes, Lynn Yang, Carsten Knutsen, Alexander Kum, Guillermina Michel, Douglas Henze, Cristina Alvira, Stephen Quake

## Abstract

Background The human myometrium undergoes an extraordinary physiological transformation during pregnancy to accommodate the growing fetus and facilitate delivery. Recent single cell RNA sequencing (scRNA-seq) studies mapping the uterine landscape, are technically limited due to underrepresentation of the large, hypertrophied uterine smooth muscle cells (uSMC) essential to pregnancy and parturition. Our high-resolution single nucleus and spatial transcriptomic atlas overcomes these constraints and provides mechanistic insights into uterine function and life-threatening complications such as uterine atony, the leading cause of postpartum hemorrhage (PPH) and global maternal mortality. Methods The present report includes comprehensive single-nucleus RNA sequencing (snRNA-seq) data of the human gravid myometrium collected during cesarean delivery from term and preterm, laboring and nonlaboring pregnant patients. Spatial validation of cell-type identities was accomplished with MERFISH and RNAscope. These data were interrogated to define: (1) pregnancy-specific smooth muscle cell signaling; (2) transcriptomic progression during labor; and (3) the molecular underpinnings of PPH due to uterine atony. Findings were validated at the RNA, protein, and functional levels in uSMC at low passage primary culture. Results Myometrial samples collected from 15 patients resolved the complex myometrial landscape into two distinct smooth muscle cell and four endothelial cell subtypes. Pregnancy induced follicle stimulating hormone receptor expression ( *FSHR)* in uSMC with definitive, functional FSH-FSHR signaling present in uSMC from the pregnant myometrium. With labor, we identified cell-type specific hypoxia signaling and metabolic reprogramming among the fibrovascular niche. We additionally discovered that patients with uterine atony upregulate expression of *ENAH* compared to those with preserved uterine contractility. ENAH is an actin-regulatory protein which orchestrates cytoskeletal dynamics; the physiologic significance of increased ENAH expression is illustrated by a distinct, stellate F-actin architecture in atony uSMC, consistent with diversion of actin into peripheral protrusions at the expense of the organized contractile apparatus required for sustained postpartum contractility. Conclusions This snRNA-seq atlas provides a foundational framework for investigation into human myometrial biology. Crucially, it characterizes previously underrepresented uterine smooth muscle cells which define the smooth muscle and endothelial cell subtypes, alongside dynamic metabolic changes in the fibrovascular niche during labor. Our work shifts the understanding of PPH from a simple failure of oxytocin signaling to, in some cases, a pre-defined mechanical state. By uncovering a pre-existing structural predisposition to uterine atony and a functional *FSHR* axis in the myometrium, this work suggests new avenues for prediction and treatment of PPH.

